# Higher Adherence to a Mediterranean Diet Is Associated with Improved Insulin Sensitivity and Selected Markers of Inflammation in Individuals Who Are Overweight and Obese without Diabetes

**DOI:** 10.3390/nu14204437

**Published:** 2022-10-21

**Authors:** Surbhi Sood, Jack Feehan, Catherine Itsiopoulos, Kirsty Wilson, Magdalena Plebanski, David Scott, James R. Hebert, Nitin Shivappa, Aya Mousa, Elena S. George, Barbora de Courten

**Affiliations:** 1 Institute for Physical Activity and Nutrition, School of Exercise and Nutrition Sciences, Faculty of Health, Deakin University, Geelong, VIC 3216, Australia; 2 Institute for Health and Sport, Victoria University, Footscray, VIC 3011, Australia; 3 Department of Medicine, Nursing and Health Sciences, School of Health and Biomedical Sciences, RMIT University, Bundoora, VIC 3083, Australia; 4 Department of Medicine, School of Clinical Sciences at Monash Health, Monash University, Clayton, VIC 3168, Australia; 5 Cancer Prevention and Control Program and Department of Epidemiology and Biostatistics, Arnold School of Public Health, University of South Carolina, Columbia, SC 29208, USA; 6 Department of Nutrition, Connecting Health Innovations LLC, Columbia, SC 29208, USA; 7 Monash Centre for Health Research and Implementation, School of Public Health and Preventive Medicine, Monash University, Clayton, VIC 3168, Australia

**Keywords:** diabetes, metabolic disease, insulin sensitivity, mediterranean diet, dietary inflammatory index, inflammation

## Abstract

Insulin resistance (IR) and chronic low-grade inflammation are risk factors for chronic diseases including type 2 diabetes (T2D) and cardiovascular disease. This study aimed to investigate two dietary indices: Mediterranean Diet Score (MDS) and Dietary Inflammatory Index (DII^®^), and their associations with direct measures of glucose metabolism and adiposity, and biochemical measures including lipids, cytokines and adipokines in overweight/obese adults. This cross-sectional study included 65 participants (males = 63%; age 31.3 ± 8.5 years). Dietary intake via 3-day food diaries was used to measure adherence to MDS (0–45 points); higher scores indicating adherence. Energy-adjusted DII (E-DII) scores were calculated with higher scores indicating a pro-inflammatory diet. IR was assessed using hyperinsulinemic euglycemic clamps, insulin secretion by intravenous glucose tolerance test, adiposity by dual-energy X-ray absorptiometry, and circulating cytokine and adipokine concentrations by multiplex assays. Higher MDS was associated with greater insulin sensitivity (β = 0.179; 95%CI: 0.39, 0.318) after adjusting for age, sex and % body fat, and lower NF-κB, higher adiponectin and adipsin in unadjusted and adjusted models. Higher E-DII score was associated with increased total cholesterol (β = 0.364; 95%CI: 0.066, 0.390) and LDL-cholesterol (β = 0.305; 95%CI: 0.019, 0.287) but not with adiposity, glucose metabolism, cytokines or adipokines. Greater MDS appears to be associated with decreased IR and inflammatory markers in overweight/obese adults.

## 1. Introduction

The prevalence of type 2 diabetes (T2D) is increasing, with an estimated 6.28% of individuals affected worldwide and over 1 million deaths attributed to diabetes each year [1]. The cost of diabetes care has also risen, accounting for 12% of global health expenditure [2]. Insulin resistance is one of the main risk factors for T2D and is implicated in multiple other chronic diseases including obesity, non-alcoholic fatty liver disease (NAFLD), polycystic ovarian syndrome, cardiovascular disease (CVD) and neurodegenerative diseases [3]. In 2020–2021, one in twenty (5.3%) people in Australia had diabetes [4] and in 2017–2018, two thirds (67.0%) of Australian adults were overweight or obese [5] with even higher rates in the USA (11.3% diabetes and 41.9% obesity [6]). We have previously shown that chronic low-grade inflammation predicts the development of insulin resistance, leading to T2D [7].

Diet is a modifiable risk factor central to the prevention and management of many chronic diseases including obesity, T2D, and CVD [8]. Earlier research has evaluated the associations between specific foods, nutrients and/or food groups, with chronic diseases [9]. More recently, anti-inflammatory diets such as the traditional Mediterranean diet (MedDiet) have emerged as having beneficial effects in obesity, T2D, and CVD in various cohorts globally [10,11]. The Mediterranean dietary pattern is habitually consumed among Mediterranean populations and is characterised by an increased intake of vegetables, legumes, fruits, nuts and unrefined cereals, a high intake of olive oil, a low-to-moderate intake of dairy products, a low intake of meat and poultry, and a moderate intake of wine during meals [12]. A high intake of fish, nuts, olive oil and red wine has been shown to reduce and protect from chronic diseases by lowering oxidative stress and inflammatory angiogenesis and improving insulin sensitivity and cardiovascular risk factors [13]. As the MedDiet is low in saturated and trans-fats, and rich in monounsaturated fatty acids, vitamins, and bioactive plant-derived phenolic compounds, with antioxidant and anti-inflammatory properties—it has been shown to lead to improvements in circulating inflammatory biomarkers [14]. Systematic reviews of dietary intervention trials have consistently shown that adopting a MedDiet results in improved glycaemic control, as well as a reduction in cardiovascular morbidity and mortality [15,16]—more so than adopting low-fat diets [17]. Additionally, the MedDiet has been associated with a greater improvement in insulin resistance in overweight/obese individuals when compared to low-fat and low-carbohydrate dietary patterns [18].

With broad anti-inflammatory properties underpinning the physiological impact of the MedDiet, measurement of the inflammatory potential of an individual’s diet has recently been of interest. The dietary inflammatory index (DII^®^) was developed as a scoring algorithm to provide an estimate for measuring the pro-inflammatory impact of diet; a high DII^®^ score reflects pro-inflammatory potential of the diet, whereas a low DII^®^ indicates the anti-inflammatory potential of diet [19]. The DII^®^ has been extensively associated with several cardiometabolic chronic diseases [20]. However, the mechanisms underpinning these associations are not yet known. To our knowledge, there are no observational studies investigating the Mediterranean diet or DII^®^ in relation to insulin sensitivity and secretion by gold standard measures such as the hyperinsulinemic euglycaemic clamp [21,22]. Only a few, small (*n* = 12) intervention studies examined the MedDiet in relation to these measures [21], and are limited to participants with existing chronic diseases such as NAFLD.

Therefore, the present study aimed to explore the associations between the Mediterranean Diet Score (MDS) and DII^®^ (Connecting Health Innovations, Columbia, SC, USA) with insulin sensitivity and secretion assessed by the hyperinsulinemic euglycaemic clamp, as well as inflammatory markers, adipokines and other cardiovascular risk factors (lipids, blood pressure), in adults without diabetes who were classified as overweight or obese.

## 2. Materials and Methods

### 2.1. Study Design and Participants

We performed a cross-sectional secondary analysis of baseline data from an interventional study, with a protocol published elsewhere [23]. Sixty-five overweight and obese, but otherwise healthy non-diabetic adults aged between 18 and 57 years were recruited from the community in Melbourne, Australia (participant flow diagram in Figure S1). The study was approved by the Monash University Human Research Ethics Committee (protocol ID: CF13/3874-2013001988, approved on 16 September 2013) and registered at clinicaltrials.gov (NCT02112721).

### 2.2. Dietary Assessments

#### 2.2.1. The Mediterranean Diet Score (MDS)

The Mediterranean dietary pattern is characterized by an increased consumption of vegetables, fruits, nuts, legumes, unprocessed cereals; a low consumption of meat and meat products; and a low consumption of dairy products. The diet also includes moderate consumption of alcohol in the form of red wine accompanied with meals. The consumption of beneficial monounsaturated fats, predominantly from olive oil and fish are recommended to the non-beneficial saturated fats [24,25]. Dietary data was collected from all participants using three-day food records. The MDS, originally described and validated by Panagiotakos et al. [26] was used and included modification of food groups to ensure it was culturally applicable and quantifiable by mapping against the Australian Guide to Healthy Eating [27] to adjust the score to suit the Australian cohort [28]. Following entry of the three-day food diaries, MDS for each participant was calculated using FoodWorks^TM^ (Xyris, Brisbane, QLD, Australia). Food intake data from three-day food diaries was re-calculated to match MDS serving sizes as described by Panagiotakos et al. [26]. Data for the food records were used to calculate nine food groups: non-refined grains, fruit, vegetables, legumes, potatoes, fish, meat and meat products, poultry, and full fat dairy products (yoghurt, cheese, milk). Olive oil and wine consumption were omitted from the total score as specific food types (e.g., red wine and extra virgin olive oil varieties) could not be accurately quantified; the approach has been used in other studies to account for these food data limitations [29,30]. Foods within the Mediterranean Dietary Pattern, including non-refined grains, fruits, vegetables, legumes, fish, and potatoes, were assigned MDS of 0 when participants reported no consumption and MDS of 1 to 5 on a scale for rare to daily consumption. Food groups that were not considered to reflect the dietary pattern, including meat and meat products, poultry, and full-fat dairy products, were assigned MDS on a reverse scale (i.e., 5 when someone reported no consumption to 0 when daily consumption was reported). Mean intake from the three-day food records was calculated and used to determine weekly consumption to calculate a MDS for each participant. For the current study, the modified overall MDS ranged from 0 to 45 [26].

#### 2.2.2. The Dietary Inflammatory Index (DII^®^)

Three-day food diaries were entered and assessed using FoodWorks™ (Xyris, Brisbane, QLD, Australia), with 28 dietary parameters derived and used to calculate the DII^®^ and the Energy-Adjusted Dietary Inflammatory Index (E-DII). Although DII consists of 45 dietary parameters, previous literature shows that predictive ability is not influenced when DII scores are calculated for 28 dietary parameters [19,31,32]. For our study, DII scores were calculated based on the following parameters: energy, protein, fat, saturated fat, trans fat, polyunsaturated fat, monounsaturated fat, cholesterol, carbohydrates, alcohol, dietary fibre, thiamine, riboflavin, niacin, vitamin C, vitamin E, vitamin B6, vitamin B12, folic acid, vitamin A, β-carotene, magnesium, iron, zinc, selenium, very long chain n-3, linoleic, caffeine. The scoring of the DII^®^ has been documented in detail elsewhere [33]. Briefly, whereby micronutrients are assigned a score per unit representation in the diet, with pro-inflammatory components, such as saturated fat and cholesterol increasing DII^®^ scores, and anti-inflammatory compounds such as fibre and B-vitamins decreasing the DII^®^. As such, a more inflammatory diet is reflected by higher scores, and an anti-inflammatory diet is reflected by lower scores. The E-DII is calculated using the same scoring system, but the scores are then adjusted for total calorie intake using an energy-adjusted global comparison database [34], to minimise the effect of food volume consumed on the final score.

### 2.3. Clinical Assessments

Anthropometric assessments included weight and height using a digital scale (Tanita BWB-600, Melbourne, VIC, Australia) and stable stadiometer (Seca 206, Melbourne, VIC Australia), respectively, from which body mass index (BMI) was calculated. Waist to hip ratio was determined from waist and hip circumference measurements, and body fat composition was assessed by dual-energy X-ray absorptiometry (DXA) (Hologic, Monash Medical Centre Radiology Department, Melbourne, VIC, Australia).

Metabolic measures included the following:

i. Oral glucose tolerance tests were conducted after a 10–12 h overnight fast. Participants ingested 75 g of glucose over 2 min. Blood samples were drawn at 0 and 120 min to analyse plasma glucose level and determine diabetes status.
ii. Hyperinsulinemic euglycemic clamps were used to measure insulin sensitivity. After collecting baseline blood and plasma glucose levels at 0 min, the clamp was initiated by an intravenous bolus injection of insulin (9 mU/kg). Insulin was then constantly infused at a rate of 40 mU/(m^2^ min) for approximately 120 min into an arm vein and glucose was variably infused to maintain euglycemia. Plasma glucose values were monitored every 5 min and the variable infusion rate of glucose was adjusted to maintain blood glucose at a constant value of 5 mmol/l for at least 30 min.
iii. Intravenous glucose tolerance tests were used to measure acute insulin secretory response. First, baseline blood was collected at −10 and 0 min, after which 50 mL of 50% glucose was delivered intravenously over a 3 min period. Blood was then collected for insulin concentration measurements at 3, 4, 5, 6, 8, 10, 15, 20, 25, and 30 min and glucose concentrations were analysed at each of these times, to determine insulin secretory response. First phase insulin secretion was calculated as the average incremental plasma insulin level from the third to the fifth minute after the glucose bolus. Second phase insulin area under the curve was calculated from the tenth to the thirtieth minute post glucose infusion.

Cardiovascular measures including blood pressure (resting systolic and diastolic), plasma lipid profile (plasma total cholesterol, triglycerides, low-density [LDL] and high-density [HDL] lipoprotein cholesterol), serum inflammatory markers (high-sensitivity C-reactive protein [hsCRP], interleukin [IL]-1β, IL-6, IL-8, IL-10, and tumor necrosis factor-alpha [TNFα]), and adipokines and chemokines (adiponectin, leptin, adipsin, resistin, and monocyte chemoattractant protein-1 [MCP-1]), as well as peripheral blood mononuclear cell concentrations of the transcription factor, NFκB, were also measured, as detailed elsewhere [23,35].

### 2.4. Statistical Methods

Statistical analysis was conducted using the Statistical Package for Social Sciences (SPSS) version 28.0.1.0 (142), (IBM, Armonk, NY, USA). Descriptive statistics for continuous data were expressed as means ± SDs or median (IQRs), while categorical data were expressed as frequencies and percentages (%). Pearson rho bivariate correlation analyses was performed to determine the relationship between MedDiet, DII^®^ and insulin resistance. R-values were classified as weak (0.10 to 0.29), moderate (0.30 to 0.49) or strong (0.50 to 1.00) correlations. Independent *t* tests were used to identify differences between groups. The assumption of normality was tested through Shapiro–Wilk tests and skewed data were transformed by natural logarithm, with adequate transformation assessed through evaluation of residuals. Multiple linear and logistic regression analyses were utilised to assess whether MDS, E-DII, DII^®^ were significant predictors for various outcome variables, adjusted for multiple confounders. Model 1 was unadjusted; Model 2 was adjusted for age and sex; Model 3 was adjusted for age, sex and % body fat; and Model 4 was adjusted for age, sex, % body fat and waist circumference. A *p*-value of <0.05 was considered significant.

## 3. Results

### 3.1. Characteristics of the Study Participants

Sixty-five individuals (41 Male/24 Female) classified as overweight (BMI ≥ 25 and <30 kg/m^2^) or obese (BMI ≥ 30 kg/m^2^) but otherwise healthy were included in the analysis. The study participants ranged in age from 18 to 57 years; and the mean age was 31.3 ± 8.5 years, with 63% males (Table 1). Mean weight was 91.8 ± 21.4 kg with a range of 59.0 to 160.0 kg, and mean BMI was 31.46 ± 5.2 kg/m^2^. Dietary data were missing for 10 participants; thus, the dietary analysis included 55 individuals. Overall, participants had a mean MDS score of 22.8 ± 4.8 out of 45.0 (range = 9.0 to 33.0). Mean MDS did not differ between sexes (males = 22.3 ± 4.7; females = 23.6 ± 4.9). Mean energy intake was 2011.0 kcal/day. Overall, mean E-DII score was 0.49 ± 1.43 and mean DII^®^ score was 0.84 ± 1.46.

### 3.2. Mediterranean Diet Score

Univariate and multivariable regression models showed associations between MDS and insulin resistance, inflammatory markers and adipokines (Table 2). Participants who achieved a higher MDS, indicated greater insulin sensitivity (mg/kg/min) after adjustment for age, sex and % body fat (β [95%CI] = 0.179 [0.39, 0.318] *p* = 0.01; Model 3, Table 2) and after adjustment for age, sex, % body fat and waist circumference (β [95%CI] = 0.180 [0.046, 0.314], *p* = 0.01; Model 4). There were no associations with other glycaemic measures (Table 2).

In relation to inflammatory markers and adipokines, higher MDS was associated with lower nuclear factor kappa-B (NF-κB) activity (β [95%CI] = −2.147; [−3.792, 0.501] *p* = 0.01), higher adiponectin (ng/mL) (β = 916.387 [265.791, 1566.983] *p* = 0.007) and adipsin (ng/mL) (β [95%CI] = 61.420 [11.893,110.948] *p* = 0.02) in the unadjusted model (Model 1), remaining significant in all adjusted models (Table 2). A higher MDS also was significantly associated with lower IL-1β (pg/mL), adjusted for age, sex, % body fat and waist circumference (β [95%CI] = 0.024 [−1.251, 1.299] *p* = 0.04; Model 4, Table 2). There were no significant associations observed between MDS and body composition, biochemical parameters (LDL, HDL, total cholesterol, and triglycerides) and other inflammatory markers or adipokines.

### 3.3. Dietary Inflammatory Index

Dietary Inflammation Index models were included as supplemental material only as E-DII was considered more appropriate for analysis since the amount of nutrients consumed is influenced by the energy value of the diet [36]. The unadjusted DII^®^ was associated with insulin secretion in both univariate and unadjusted models, but none of the other variables (Table S1).

The E-DII was not associated with body fat composition or glycaemic parameters before or after adjustments (Table 3). Higher E-DII score was associated with higher total cholesterol (mmol/L) in Model 1 (β [95%CI] = 0.364 [0.066, 0.390] *p* = 0.007), Model 2 (β = 0.351 [0.069, 0.370] *p* = 0.005) and Model 3 (β [95%CI] = 0.347 [0.068, 0.365] *p* = 0.005). Higher E-DII was also associated with higher LDL-cholesterol (mmol/L) in Model 1 (β [95%CI] = 0.305 [0.019, 0.287] *p* = 0.03), Model 2 (β [95%CI] = 0.293 [0.019, 0.275] *p* = 0.03) and model 3 (β [95%CI] = 0.291 [0.018, 0.274] *p* = 0.03). There were no associations with inflammatory markers or adipokines (all *p* > 0.05).

## 4. Discussion

In the present study, we investigated whether the MDS and/or the DII^®^ score was associated with measures of body composition, insulin resistance and secretion, blood pressure, lipids, cytokines and adipokines in individuals who were either overweight or obese without diabetes. To the best of our knowledge, this is the first study to explore the relationships between MDS, DII^®^ and gold-standard measures (hyperinsulinemic euglycaemic clamp), of insulin sensitivity and secretion in adults who were overweight or obese, without diabetes.

We showed that higher MDS was associated with greater insulin sensitivity and improved inflammation. Energy-adjusted DII was associated with total cholesterol and LDL cholesterol, with no significant associations found with body composition, insulin sensitivity and secretion, inflammatory markers or adipokines. This is novel given the fact that over 40 published studies have observed associations between the DII^®^/E-DII and at least one marker of inflammation, however, these were predominantly in individuals with chronic non-communicable diseases. Furthermore, the E-DII scores herein were not very high compared to other studies.

**Figure 1:**
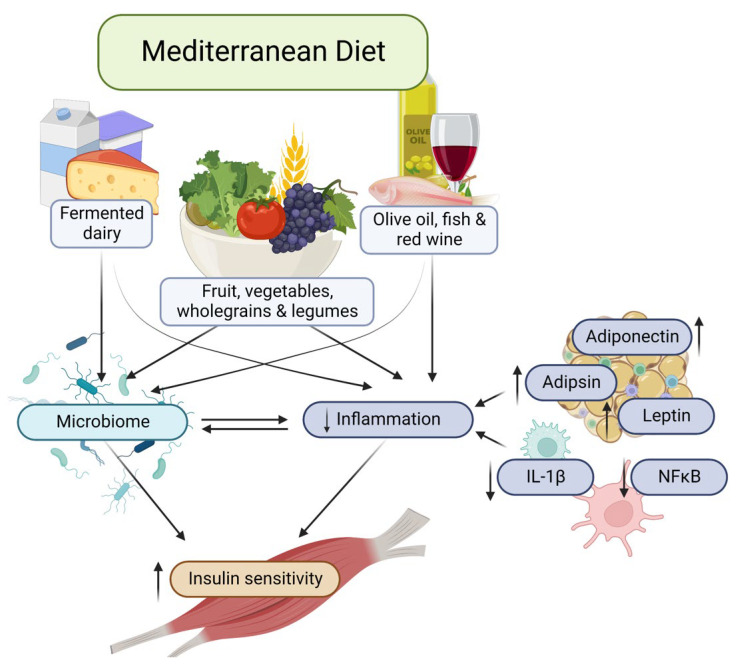
Principal mechanisms responsible for playing a role in protecting against insulin resistance and inflammation regulated by Mediterranean diet.

The associations of MDS with insulin resistance and inflammation have been investigated in cross-sectional and interventional studies [37,38], with several putative mechanisms thought to underpin these relationships. Schematic representation of the principal mechanisms responsible for playing a role protecting against insulin resistance and inflammation regulated by MedDiet are demonstrated in Figure 1.

Our findings of an association between MDS and insulin sensitivity are consistent with studies using less robust measures to quantify insulin resistance. For instance, the ATTICA study with 3042 adult males and females in Greece, who assessed insulin sensitivity using the Homeostatic Model Assessment of insulin resistance (HOMA-IR) approach, reported modest correlations between HOMA-IR and lower adherence to the MedDiet in individuals classified as overweight and obese [37]. In contrast, our study included a comparably low MDS, which was not unexpected in a non-Mediterranean cohort (mean score 22.8 ± 4.8 out of 45 vs. the ATTICA study where >48% of participants were in the third tertile indicating > 24.0 out of 55) [37]. Additionally, in agreement with our findings, the Multi-Ethnic Study of Atherosclerosis (MESA), a cohort study conducted in the United States with 6814 participants free of diabetes and CVD, reported improved insulin sensitivity by HOMA-IR in participants who had higher adherence to a Mediterranean-style diet [39]. Our study did not observe any associations with MedDiet and insulin secretion. A previous randomised controlled trial conducted in Italy with 12 healthy adults without diabetes, investigated the effectiveness of MedDiet vs. Zone Diet (centred on protein intake) on overall insulin sensitivity and secretion (measured by oral glucose insulin sensitivity index and C-peptide data, respectively); suggesting that both diets did not result in significant changes in insulin sensitivity and secretion from baseline [40]. Of note is that previous studies have been predominantly conducted in Mediterranean countries with higher MedDiet consumption and using indirect measures of insulin sensitivity and secretion.

Chronic low-grade inflammation, characterised by elevated pro-inflammatory and/or reduced anti-inflammatory cytokine or adipokine concentrations, has been shown to predict obesity, insulin resistance, T2D, metabolic syndrome and CVD [7,41,42]. We showed that participants with greater MDS indicated reduced NF-κB activity in peripheral blood mononuclear cells, reduced circulating inflammatory markers (IL-1β) and increased adipokines (adiponectin and adipsin), findings that are in line with other literature [43,44]. The ATTICA study reported that greater adherence to the MedDiet was independently associated with lower markers of inflammation and coagulation including CRP and IL-6 [45]. The PREDIMED sub-study conducted in Spain, using data collected from 285 participants at risk of coronary heart disease, reported a reduction in plasma levels of IL-1β, consistent with our study [46]. This improvement in inflammation can be explained by the high polyphenol content, a key component of the MedDiet, which is known to inhibit proinflammatory markers such as IL-1β and NF-κB activation [47].

In previous literature, adiponectin, an adipose tissue–secreted cytokine, has been reported to show improvements in insulin sensitivity, glucose regulation, lipid metabolism, and reduce atherosclerosis [48]. In a previous study including 598 participants aged 12–65 years old of the Balearics Islands (a Mediterranean region), higher adherence to the MedDiet in adults was associated with higher levels of adiponectin, but not in young adult participants (12–17 years) [44]. In addition, the Nurses’ Health Study, a cross-sectional study with 121,700 females aged between 30 and 55 years, reported that higher adherence to MedDiet was associated with higher adiponectin independent of adiposity [43]. Similarly, adipsin (also known as complement factor D), is a major protein of adipose cells and is recognised as a key requirement for proper insulin secretion by pancreatic β cells [49]. Mice lacking adipsin exhibit worsened glucose homeostasis in states of metabolic stress caused by diet-induced obesity and previous reports demonstrate the dysregulation of adipsin in models of obesity and diabetes [50,51]. In the present study, participants who achieved a greater MDS, presented with higher adipsin, which is consistent with the single previous study in this area [52], where adipsin was increased in response to both exercise and a MedDiet intervention in adults with no underlying medical conditions. In contrast, fast foods with high saturated or total fat content have been shown to reduce adipsin concentrations [52,53], establishing adipsin as a potentially important link between diet and obesity/energy homeostasis, systemic metabolism and immunoregulation.

Most individuals with T2D are classified as overweight or obese (~80%) and thus have high rates of increased waist circumference, visceral adiposity, and total body fat. Visceral fat has a detrimental impact on insulin sensitivity [54] as adipose tissue is linked with unfavourable secretion of hormones and pro-inflammatory chemicals, glycerol, leptin, cytokines, and lower secretion of adiponectin exacerbating insulin resistance. Abdominal fat does not respond well to the antilipolytic action of insulin, leading to insulin resistance and therefore T2D [55]. In this cohort of individuals with overweight and obesity, some of these adverse health outcomes associated with adiposity may be moderated by consuming a healthy MedDiet as a result of its anti-inflammatory properties. There was no relationship with direct measures of adiposity, measured by DXA, which is likely due to the small sample size in our study, and the population comprising only individuals with overweight and obesity without a lean comparison group. Furthermore, visceral adiposity measures (which were not assessed in this study), might have been associated with MedDiet.

Given the anti-inflammatory rationale underpinning the known benefits of the MedDiet, we hypothesized that similar associations would be found between DII^®^, an indicator of pro-inflammatory diet, and cardiometabolic measures. However, we found no associations except of E-DII with total and LDL-cholesterol, which is consistent with a previous study reporting that higher E-DII scores predict higher LDL-cholesterol [56]. Another study reported that a higher E-DII score was positively correlated with LDL and total cholesterol, and inversely with HDL-cholesterol [57]. However, previous research using the DII^®^ and dyslipidaemia have been mixed, with some finding no relationship between DII and lipids in individuals with chronic diseases, likely due to some individuals taking hypolipidemic medications, making these results challenging to decipher [20]. We did not find associations between E-DII and insulin sensitivity or secretion; however, this is the first study using hyperinsulinemic euglycaemic clamp to assess these outcomes and further confirmation in larger cohorts is needed. To date, DII^®^ scores have been associated with an increased risk of obesity and CVD, but the relationship with T2D has been largely conflicting [20]. While the reasons underpinning the disparity are not known, one potential cause is the varied methodologies for assessment of these outcomes and populations across studies and the inability to directly compare results, particularly with studies using indirect proxy glycaemic measures such as HOMA [20]. Moreover, the DII^®^ is a literature-derived score based on a varying level of evidence from six different inflammatory markers (two of which are anti-inflammatory) and is therefore an indirect measure of inflammation with associated limitations.

The MedDiet has been shown to elicit health benefits including improvements in metabolic risk factors due to its anti-inflammatory composition as well as its antioxidant actions. Another factor that has been explored throughout the literature, albeit not directly assessed herein, but may provide some insight into the proposed benefits of the dietary pattern is the gut microbiome. Indeed, the MedDiet is known to improve the composition and health of the microbiome, due to its high content of pre- and pro-biotic fibre, starches, and nutrients, though these components of the dietary pattern are not explicitly captured within the DII [58]. These beneficial effects of the MedDiet on the microbiome are thought to attenuate, at least in part, both the anti-inflammatory and other positive metabolic outcomes associated with the diet [59,60]. Moreover, the anti-inflammatory plant-rich dietary components of the MedDiet are hypothesised to have benefits that are observed regardless of weight [28].

### Strengths and Limitations

An important strength of the present this study is that unlike in previous studies [37,61], obesity, insulin resistance, and secretion were measured by gold-standard techniques. This study contributes to the limited literature that even with a generally lower MDS in a non-Mediterranean cohort, those with higher MDS had lower insulin resistance and chronic low-grade inflammation. This suggests that the MedDiet has the potential to slow or ameliorate progression to T2D, although this association requires further confirmation by clinical trials. The current study, however, has some limitations. The sample size was moderate, and the cross-sectional design precludes causal inferences. The use of food diaries is also limited due to potential errors with dietary recall and social desirability bias, which can lead to inaccurate or underreporting. The MDS was modified to account for data availability, where oil type and alcohol consumption could not be accurately quantified, hence the potential contribution to MDS and benefits of these dietary components could not be assessed. Transferability of a Mediterranean dietary pattern in non-Mediterranean regions is complex, as seen in previous literature and, as such, adherence indicated by MDS was low for participants herein, providing a limited score range for interpretation [28]. Additionally, future large-scale studies may provide further insights into whether a higher or lower level of adherence to the MedDiet drives improvements in health outcomes. Notwithstanding these limitations, our findings suggest potentially clinically meaningful benefits despite a lower overall MDS in this non-Mediterranean population.

## 5. Conclusions

In conclusion, a greater MDS was associated with higher insulin sensitivity measured by hyperinsulinemic-euglycemic clamp, and better inflammation profile in adults who are overweight or obese. Energy-adjusted DII was associated with total cholesterol and LDL. Future studies including longitudinal and interventional study designs with larger sample sizes are warranted to confirm these findings and determine whether MedDiet can be an effective management strategy to improve insulin sensitivity and inflammation and slow or ameliorate associated chronic conditions in non-Mediterranean cohorts.

## Figures and Tables

**Table 1:** Lifestyle, clinical and biochemical baseline characteristics of the study participants ^1^.

Characteristics	Overall (*n* 65)	Males (*n* 41)	Females (*n* 24)
	Mean ± SD		
Anthropometry parameters			
Weight (kg)	91.8 ± 21.4	93.2 ± 20.0	89.4 ± 23.9
Height (cm)	169.0 ± 9.9	174.0 ± 7.7	160.5 ± 7.2
BMI (kg/m^2^)	31.5 ± 5.2	30.5 ± 4.8	33.0 ± 5.7
Hip circumference (cm)	111.1 ± 11.2	108.6 ± 9.7	115.5 ± 12.3
Waist circumference (cm)	123.4 ± 158.8	134.8 ± 199.7	104.1 ± 13.9
Body fat (%) *	40.1 ± 8.7	35.2 ± 6.0	48.8 ± 5.4
Fat mass (%) *	36.0 ± 11.0	33.5 ± 11.8	40.5 ± 7.8
Fat free mass *	53.4 ± 12.4	59.8 ± 10.3	42.0 ± 5.8
Mediterranean Diet Score (MDS)			
Score **	22.8 ± 4.8	22.3 ± 4.7	23.6 ± 4.9
Energy-Adjusted Dietary Inflam- matory Index (E-DII)			
Score ***	0.49 ± 1.43	0.52 ± 1.62	0.44 ± 1.04
Dietary Inflammatory Index (DII)			
Score ***	0.84 ± 1.46	0.83 ± 1.48	0.86 ± 1.5
Socio-demographic characteristics			
Age, mean years (years)	31.3 ± 8.5	31.2 ± 8.4	31.6 ± 8.7
Ethnicity (%)			
Caucasian	19	10	9
South/Central Asia	21	14	7
South/East and North/East Asia	15	10	5
Other	7	5	2
Glucose metabolism and insulin sensitivity parameters			
Fasting blood glucose (mmol/L)	4.6 ± 0.5	4.7 ± 0.5	4.4 ± 0.5
2 h blood glucose OGTT (mmol/L)	5.6 ± 1.6	5.5 ± 1.6	5.7 ± 1.7
M-value for insulin sensitivity from clamp (mg/kg/min)	6.6 ± 2.8	7.0 ± 3.0	5.9 ± 2.4
Fasting Insulin (mIU/L)	10.4 ± 6.2	10.3 ± 6.3	10.7 ± 6.1
Insulin AUC (0–30 min) (mlU/L)	1972.2 ± 1302.1	1987.2 ± 1369.0	1946.5 ± 1207.1
First phase insulin AUC (3–5 min post glucose infusion) (mlU/L)	385.0 ± 291.7	379.7 ± 300.4	394.1 ± 282.4
Second phase insulin AUC (10–30 min post glucose infusion) (mlU/L)	1303.6 ± 856.8	1310.0 ± 892.4	1292.8 ± 810.9
Haemodynamic parameters			
SBP (mmHg)	121.0 ± 12.5	124.7 ± 12.1	114.9 ± 10.7
DBP (mmHg)	80.3 ± 8.6	80.0 ± 9.2	80.7 ± 7.6
Heart rate (bpm)	75.3 ± 11.7	74.5 ± 13.1	76.6 ± 8.8
Biochemical parameters			
Total cholesterol (mmol/L)	5.0 ± 0.9	5.1 ± 1.0	4.6 ± 0.7
Triglycerides (mmol/L)	1.6 ± 0.9	1.8 ± 1.0	1.2 ± 0.4
HDL (mmol/L) *	1.2 ± 0.3	1.2 ± 0.2	1.2 ± 0.3
LDL (mmol/L) *	3.1 ± 0.7	3.2 ± 0.8	2.9 ± 0.6
Inflammatory Markers			
Adiponectin (ng/mL)	9306.0 ± 12196.3	7499.7 ± 8123.4	12391.7 ± 16834.3
Leptin (ng/mL)	14.8 ± 28.5	4.7 ± 7.4	32.1 ± 40.9
Adipsin (ng/mL)	1058.2 ± 914.7	987.8 ± 792.4	1178.5 ± 1101.0
Resistin (ng/mL)	0.9 ± 1.2	0.8 ± 1.3	1.0 ± 0.8
High-sensitivity C-reactive protein (mg/L) *	3.3 ± 3.8	3.1 ± 4.2	3.6 ± 3.0
TNFα (pg/mL)	52.9 ± 57.0	41.6 ± 39.5	72.2 ± 75.5
MCP1 (pg/mL)	913.7 ± 813.1	781.5 ± 541.7	1139.6 ± 1115.8
IL6 (pg/mL)	39.0 ± 44.3	29.4 ± 27.3	55.3 ± 61.0
IL10 (pg/mL)	12.7 ± 10.6	11.7 ± 10.5	14.5 ± 10.6
IL-1β (pg/mL)	26.8 ± 23.2	22.1 ± 19.5	34.7 ± 27.1
IL8 (pg/mL) *	18.5 ± 14.3	15.8 ± 10.3	23.0 ± 18.5

^1^ Values are means ± SDs, medians (IQR) or frequencies (percentages). Abbreviations: M-value for insulin sensitivity from clamp, SBP; systolic blood pressure, DBP; diastolic blood pressure, HDL; high-density lipoprotein, LDL; low density lipoprotein, CRP, c-reactive protein; IPAQMETS; International physical activity questionnaire; TNFa, tumor necrosis factor-alpha; MCP, monocyte chemoattractant protein-1; IL6, interleukin-6; IL10, interleukin-10; IL18, interleukin-18; IL1B, interleukin-1 beta; IFNa, interferon alpha; IFNg, interferon gamma; IL8, interleukin-8; IL12, interleukin-12; IL17, interleukin-17; IL23, interleukin-23; IL33, interleukin-33 * Overall *n* = 64 for Body fat, Fat mass, Fat-free mass, HDL, LDL, CRP, IL8, IL23, Physical activity METS; Males *n* = 41 for HDL, LDL, CRP, IL8, IL23; Females *n* = 23 for Body fat, Fat mass, Fat-free mass, Physical activity METS. ** MDS *n* = 55. *** E-DII *n* = 48. Caucasian: Australia, NZ, UK, Irish, American; South/Central Asia: India, Sri Lanka, Pakistan, Afghan, Bangladesh; South/East and North/East Asia: China, Japan, Korea, Taiwan, Malaysia, Indonesia, Thailand, Singapore; Other: Africa, Middle East, Polynesia, South America.

**Table 2:** Associations between Mediterranean Diet Score and anthropometry data, insulin resistance, and inflammatory markers (*n* = 55).

Parameters	Beta Coefficients, 95% Confidence Interval and *p*-Value							
Models	Model 1 ^a^		Model 2 ^b^		Model 3 ^c^		Model 4 ^d^	
*p*-Value	β (%95 CI)	*p*	β (%95 CI)	*p*	β (%95 CI)	*p*	β (%95 CI)	*p*
Glucose metabolism and insulin sensitivity parameters								
Fasting blood glucose (mmol/L)	−0.012 (−0.041, 0.018)	0.43	−0.008 (−0.037, 0.022)	0.60	−0.007 (−0.037, 0.022)	0.62	−0.008 (−0.038, 0.022)	0.61
2 h blood glucose OGTT (mmol/L)	0.009 (−0.085, 0.102)	0.86	0.004 (−0.092, 0.025)	0.94	−0.001 (−0.097, 0.096)	0.99	−0.001 (−0.098, 0.097)	0.99
M-value for insulin sensitivity from clamp (mg/kg/min)	0.127 (−0.031, 0.284)	0.11	0.142 (−0.015, 0.300)	0.08	0.179 (0.39, 0.318)	0.01 *	0.180 (0.046, 0.314)	0.01 *
Biochemical parameters								
Total cholesterol (mmol/L)	−0.020 (−0.071, 0.032)	0.45	−0.015 (−0.065, 0.035)	0.54	−0.011 (−0.062, 0.39)	−0.46	−0.12 (−0.059, 0.035)	0.61
Triglycerides (mmol/L)	−0.011 (−0.060, 0.038)	0.65	−0.002 (−0.048, 0.045)	0.95	0.001 (−0.046, 0.048)	0.96	0.001 (−0.046, 0.048)	0.96
HDL (mmol/L)	0.000 (−0.015, 0.014)	0.98	−0.002 (−0.016, 0.013)	0.81	−0.001 (−0.016, 0.014)	0.88	−0.001 (−0.016, 0.014)	0.87
LDL (mmol/L)	−0.016 (−0.058, 0.026)	0.45	−0.014 (−0.056, 0.028)	0.50	−0.012 (−0.055, 0.030)	0.57	−0.012 (−0.053, 0.028)	0.54
Inflammatory Markers								
Adiponectin (ng/mL)	916.387 (265.791, 1566.983)	0.007 *	853.284 (195.429, 1511.138)	0.01 *	854.383 (184.113, 1524.653)	0.01 *	857.071 (186.498, 1527.644)	0.01 *
Leptin (ng/mL)	1.571 (−0.003, 3.144)	0.05 *	1.234 (−0.213, 2.680)	0.09	1.053 (−0.365, 2.472)	0.14	1.045 (−0.361, 2.452)	0.14
Adipsin (ng/mL)	61.420 (11.893, 110.948)	0.02 *	57.377 (8.534, 106.221)	0.02 *	55.476 (5.888, 105.063)	0.03 *	55.569 (5.563, 105.576)	0.03 *
NF-κB	−2.147 (−3.792, −0.501)	0.01 *	−2.139 (−3.840, −0.438)	0.02 *	−2.342 (−4.008, −0.676)	0.007 *	−2.345 (−4.029, −0.660)	0.008 *
TNFα (pg/mL)	0.567 (−2.689, 3.824)	0.73	0.301 (−2.844, 3.446)	0.85	0.577 (−2.569, 3.722)	0.71	0.586 (−2.573, 3.746)	0.71
IL-1β (pg/mL)	0.031 (−1.297, 1.359)	0.96	−0.071 (−1.330, 1.187)	0.91	0.021 (−1.245, 1.287)	0.97	0.024 (−1.251, 1.299)	0.04

Beta coefficients represent change in outcome measure per 1-unit increase in Mediterranean Diet Score; * significant at *p* < 0.05. ^a^ Model 1: Unadjusted; ^b^ Model 2: Adjusted for age and sex; ^c^ Model 3: Adjusted for age, sex, % body fat; ^d^ Model 4: Adjusted for age, sex, % body fat and waist circumference. Abbreviations: NF-κB, Nuclear factor kappa B; IL-1β, Interleukin 1 beta; TNFα, Tumor necrosis factor-alpha.

**Table 3:** Associations between Energy Adjusted Dietary Inflammatory Index and anthropometry data, insulin resistance and biochemical parameters (*n* = 55).

Parameters	Beta Coefficients, 95% Confidence Interval and *p*-Value							
Models	Model 1 ^a^		Model 2 ^b^		Model 3 ^c^		Model 4 ^d^	
*p*-Values	β (%95 CI)	*p*	β (%95 CI)	*p*	β (%95 CI)	*p*	β (%95 CI)	*p*
Glucose metabolism and insulin sensitivity parameters								
Fasting blood glucose (mmol/L)	−0.043 (−0.116, 0.085)	0.76	−0.046 (−0.116, 0.082)	0.74	−0.046 (−0.117, 0.083)	0.74	−0.040 (−0.103, 0.074)	0.75
2 h blood glucose OGTT (mmol/L)	−0.182 (−0.091, 0.018)	0.19	−0.182 (−0.090, 0.018)	0.19	−0.183 (−0.091, 0.019)	0.89	−0.177 (−0.086, 0.016)	0.17
M-value for insulin sensitivity from clamp (mg/kg/min)	−0.020 (−0.098, 0.085)	0.88	−0.029 (−0.100, 0.080)	0.83	−0.037 (−0.197, 0.073)	0.77	−0.043 (−0.089, 0.060)	0.70
Biochemical parameters								
Total cholesterol (mmol/L)	0.364 (0.066, 0.390)	0.007 *	0.351 (0.069, 0.370)	0.005 *	0.347 (0.068, 0.365)	0.005 *	0.348 (0.068, 0.367)	0.005 *
Triglycerides (mmol/L)	0.179 (−0.032, 0.154)	0.20	0.171 (−0.030, 0.145)	0.19	0.167 (−0.030, 0.143)	0.20	0.169 (−0.030, 0.144)	0.19
HDL (mmol/L)	0.102 (−0.032, 0.068)	0.47	0.101 (−0.031, 0.068)	0.46	0.101 (−0.032, 0.068)	0.47	0.099 (−0.033, 0.068)	0.48
LDL (mmol/L)	0.305 (0.019, 0.287)	0.03 *	0.293 (0.019, 0.275)	0.03 *	0.291 (0.018, 0.274)	0.03 *	0.292 (0.017, 0.276)	0.03 *

Beta coefficients represent change in outcome measure per 1-unit increase in E-DII; * significant at *p* < 0.05. ^a^ Model 1: Unadjusted; ^b^ Model 2: Adjusted for age and sex; ^c^ Model 3: Adjusted for age, sex, % body fat; ^d^ Model 4: Adjusted for age, sex, % body fat and waist circumference. Abbreviations: LDL, low density lipoprotein; HDL, high density lipoprotein.

## Data Availability

Data described in the manuscript, code book, analytic code will not be made available due to privacy and ethical reasons.

## Supplementary Materials

The following supporting information can be downloaded at: https://www.mdpi.com/article/10.3390/nu14204437/s1, Figure S1: Flow diagram of subjects who participated in the study according to the STROBE statement; Table S1: Associations between Dietary Inflammatory Index and insulin resistance, anthropometry data, and metabolic factors (*n* = 55).
