# Supplementary material for: Higher Adherence to a Mediterranean Diet Is Associated with Improved Insulin Sensitivity and Selected Markers of Inflammation in Individuals Who Are Overweight and Obese without Diabetes
Source: Nutrients. 2022 Oct 21;14(20):4437. doi: 10.3390/nu14204437 (PMC9608711; doi:10.3390/nu14204437)
Supplement: Supplementary file 1 [file nutrients-14-04437-s001.zip › nutrients-1979763-supplementary.pdf]

Supplementary Materials

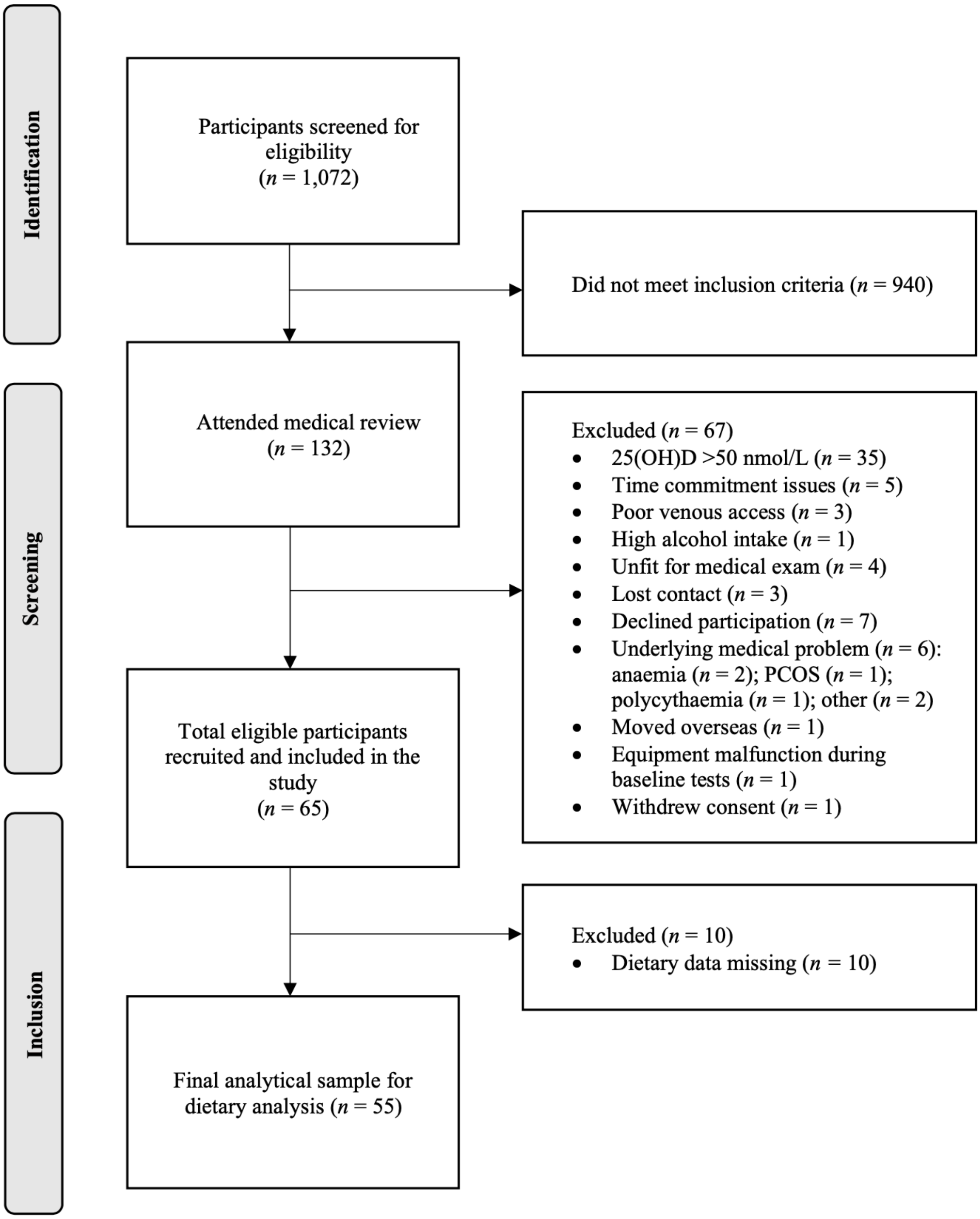

Figure S1. Flow diagram of subjects who participated in the study according to the STROBE statement [1].

Table S1. Associations between Dietary Inflammatory Index and insulin resistance, anthropometry data, and metabolic factors (n=55).

Parameters	Beta Coefficients, 95% Confidence Interval and *P*-value							
	Model 1[a]		Model 2[b]		Model 3[c]		Model 4[d]	
	β (%95 CI)	*p*	β (%95 CI)	*p*	β (%95 CI)	*p*	β (%95 CI)	*p*
Weight (kg)	−0.168 (−3.126, 0.750)	0.22	−0.163 (−0.054, 0.014)	0.24				
BMI (kg/m2)	−0.124 (−1.194, 0.453)	0.37	−0.127 (−2.426, 0.423)	0.35				
Hip circumference (cm)	−0.127 (−2.510, 0.924)	0.36	−0.131 (−2.426, 0.796)	0.32				
Waist circumference (cm)	−0.032 (−0.077, 0.061)	0.82	0.006 (−3.164, 0.791)	0.23				
Body fat (%)	−0.054 (−0.022, 0.015)	0.70	−0.061 (−0.017, 0.008)	0.51				
Fat mass (%)	−0.094 (−0.075, 0.037)	0.50	−0.098 (−0.070, 0.030)	0.43				
Fat free mass	−0.066 (−0.050, 0.031)	0.64	−0.058 (−0.035, 0.018)	0.53				
**Glucose metabolism and insulin sensitivity parameters**								
Fasting blood glucose (mmol/L)	0.115 (−0.057, 0.139)	0.41	0.118 (−0.054, 0.138)	0.39	0.118 (−0.056, 0.139)	0.62	0.118 (−0.044, 0.128)	0.34
2-hour blood glucose OGTT (mmol/L)	−0.159 (−0.084, 0.023)	0.25	−0.160 (−0.085, 0.022)	0.25	−0.164 (−0.086, 0.022)	0.24	−0.164 (−0.082, 0.018)	0.22
M-value for insulin sensitivity from clamp (mg/kg/min)	0.055 (−0.072, 0.107)	0.69	0.058 (−0.069, 0.106)	0.67	0.024 (−0.075, 0.092)	0.84	0.026 (−0.065, 0.082)	0.82
Fasting Insulin at baseline (mIU/L)	−0.332 (−0.210, -0.025)	0.01*	−0.333 (−0.209, -0.026)	0.01*	−0.326 (−0.208, −0.023)	0.02*	−0.326 (−0.208, −0.022)	0.02*
Insulin Area under the curve (total from 0-30 min) at baseline (mlU/L)	−0.343 (−0.290, −0.039)	0.01*	−0.342 (−0.290, −0.039)	0.01*	−0.343 (−0.292, −0.037)	0.01*	−0.343 (−0.293, −0.036)	0.01*
First phase insulin area under the curve (minutes 3-5 post glucose infusion) at baseline (mlU/L)	−0.329 (−0.360, −0.041)	0.02*	−0.330 (−0.361, −0.040)	0.02*	−0.337 (−0.367, −0.043)	0.01*	−0.337 (−0.366, −0.044)	0.01*
Second phase insulin area under the curve (minutes 10-30 post glucose infusion) at baseline (mlU/L)	−0.321 (−0.269, −0.027)	0.02*	−0.321 (−0.269, −0.026)	0.02*	−0.316 (−0.269, −0.022)	0.02*	−0.316 (−0.270, −0.021)	0.02*
**Haemodynamic parameters**								

SBP (mmHg)	−0.010 (−2.494, 2.320)	0.94	−0.007 (−2.197, 2.080)	0.96	0.010 (−2.046, 2.211)	0.94	NS
DBP (mmHg)	0.060 (−1.304, 2.015)	0.67	0.059 (−1.250, 1.951)	0.66	0.073 (−1.169, 2.040)	0.59	NS
Heart rate (bpm)	−0.012 (−2.302, 2.104)	0.93	−0.015 (−2.298, 2.061)	0.91	−0.007 (−2.255, 2.151)	0.96	NS
Pulse pressure (systolic - diastolic blood pressure) (mmHg)	−0.069 (−2.225, 1.341)	0.62	−0.064 (−1.941, 1.123)	0.59	−0.055 (−1.899, 1.193)	0.65	NS
Mean Arterial Pressure (diastolic x2 + systolic)/3 (mmHg)	0.033 (−1.541, 1.958)	0.81	0.034 (−1.432, 1.862)	0.80	0.051 (−1.324, 1.960)	0.70	NS
**Biochemical parameters**							
Total cholesterol (mmol/L)	0.035 (−0.149, 0.192)	0.80	0.038 (−0.136, 0.183)	0.77	0.020 (−0.146, 0.171)	0.46	NS
Triglycerides (mmol/L)	−0.028 (−0.102, 0.083)	0.84	−0.024 (−0.095, 0.079)	0.85	−0.042 (−0.101, 0.073)	0.75	NS
HDL (mmol/L)	−0.015 (−0.053, 0.048)	0.92	−0.024 (−0.054, 0.046)	0.86	−0.026 (−0.056, 0.046)	0.85	NS
LDL (mmol/L)	0.658 (−0.095, 0.026)	0.51	0.094 (−0.087, 0.181)	0.49	0.081 (−0.095, 0.176)	0.55	NS

Beta coefficients represent change in outcome measure per 1-unit increased in DII; *significant at $p<0.05$. [a] Model 1: Unadjusted; [b] Model 2: Adjusted for age and sex; [c] Model 3: Adjusted for age, sex, % body fat; [d] Model 4: Adjusted for age, sex, % body fat and waist circumference.
